# The role of organ-deposited IgG in the pathogenesis of multi-organ and tissue damage in systemic lupus erythematosus

**DOI:** 10.3389/fimmu.2022.924766

**Published:** 2022-10-13

**Authors:** Wenlin Qiu, Tong Yu, Guo-Min Deng

**Affiliations:** Department of Rheumatology and Immunology, Union Hospital, Tongji Medical College, Huazhong University of Science and Technology, Wuhan, China

**Keywords:** SLE, organ damage, autoantibody, macrophage, IgG, FcγR

## Abstract

Systemic lupus erythematosus (SLE), often known simply as lupus, is a severe chronic autoimmune disease that is characterized by multi-organ and tissue damage and high levels of autoantibodies in serum. We have recently investigated, using animal models, the role of organ-deposited IgG autoantibodies in the pathogenesis of organ and tissue damage in SLE. We found that intra-organ injection of serum from mice with lupus (i.e., lupus mice) into healthy mice triggered inflammation in tissue and organs but that serum from other healthy mice did not, and that the severity of inflammation was related to the dose of serum injected. Immunohistochemistry showed that a large number of IgG molecules are deposited at the site of organ and tissue damage in lupus mice, and that IgG is a major contributor to the development of tissue inflammation triggered by serum from lupus mice or patients. The development of tissue inflammation induced by IgG in serum from lupus mice requires the presence of monocytes/macrophages, but not of lymphocytes or neutrophils; tumor necrosis factor (TNF)/tumor necrosis factor receptor 1 (TNFR1) and interleukin 1 (IL-1) also play essential roles in the development of tissue inflammation triggered by IgG. In addition, it has been found that TNFR1 inhibitors can suppress skin injury in lupus mice and that spleen tyrosine kinase (Syk) inhibitors, which can block the signaling transduction of IgG/Fc gamma receptors (FcγRs), can prevent and treat skin injury and kidney damage in lupus mice. We have also observed that lupus IgG might protect against bone erosion. Based on these results, we conclude that IgG plays a crucial role in the development of organ and tissue damage in SLE and in protecting bone erosion and arthritis, and we suggest that the IgG/FcγR signaling pathway is an important therapeutic target in SLE.

## Introduction

Systemic lupus erythematosus (SLE), which affects mostly women of childbearing age, is a chronic severe autoimmune disease characterized by damage to multiple organs and tissues and a high level of autoantibodies in serum (1–3). The incidence and prevalence of SLE vary considerably between different regions of the world (1, 4). In adults, the prevalence of SLE worldwide ranges from 30 to 150 per 100,000, and the incidence ranges from 2.2 to 23.1 per 100,000 per year (4). The typical initial symptoms of lupus are fever, erythema, and arthritis (5). Renal involvement is the most common manifestation: the prevalence of nephritis among lupus patients is 29–82% (6). Skin injury is the second most common symptom, and occurs in 70–85% of all lupus patients (7). Arthralgia is also common, and lupus patients with arthralgia often suffer from varying degrees of synovitis, but without bone erosion (8). Only 2.8–4.3% of patients have Jaccoud’s arthropathy, which may result in joint deformities (8). With advancements in therapies, 5-, 10-, 15-, and 20-year survival rates have risen to 95%, 91%, 85%, and 78%, respectively (4). The main causes of death in SLE patients are infection, cardiovascular events, and active disease (4, 9). Glucocorticoids and antimalarial agents (especially hydroxychloroquine) are the most important and most common first-line agents for the treatment of SLE, but B-cell-targeting drugs have shown promising results (4, 5). Belimumab, a humanized monoclonal antibody targeting soluble B-cell activating factor (BAFF), a B-cell stimulator, can significantly and sustainedly reduce the level of IgG autoantibodies, including anti-double-stranded DNA (anti-dsDNA), anti-Smith (anti-Sm), anti-cardiolipin, and anti-ribosomal P autoantibodies (10). In addition, the immature dendritic cell (iDC) vaccine, an underdeveloped therapy, has been shown to have a protective effect in lupus-like nephritis induced by pathogenic autoantibodies (11). These indicates that SLE IgG is involved in the therapy of SLE.

The organ and tissue damage that occurs in the region of IgG deposition, is due to infiltration by inflammatory cells, leading to the destruction of tissue organization. Severe damage to multiple organs is one of the leading causes of death in patients with SLE (3, 12). Commonly damaged organs include the kidneys, skin, joints, liver, spleen, lungs, and brain (1–3).

The immune system is damaged in SLE patients, and B cells are abnormally over activated and produce multiple types of autoantibodies. Patients with SLE produce IgA, IgE, IgG, and IgM autoantibodies (12), but IgG autoantibodies predominate. Inflammation is the pathological basis of organ and tissue damage caused by the local deposition of IgG and immune complexes (ICs) (12–17). The pathogenesis of SLE is closely associated with a high level of autoreactive IgG (2, 3, 18, 19). However, the mechanism by which IgG causes organ and tissue damage is still unclear.

Tissue-deposited ICs formed by autoantibodies and autoantigens activate immune cells to produce inflammatory cytokines by binding to Fc gamma receptors (FcγRs) (20–24). Recently, we have demonstrated that organ-deposited IgG induces local inflammation by binding to FcγRs on the surface of monocytes/macrophages, resulting in multi-organ and tissue damage (20–28).

**Figure 1:**
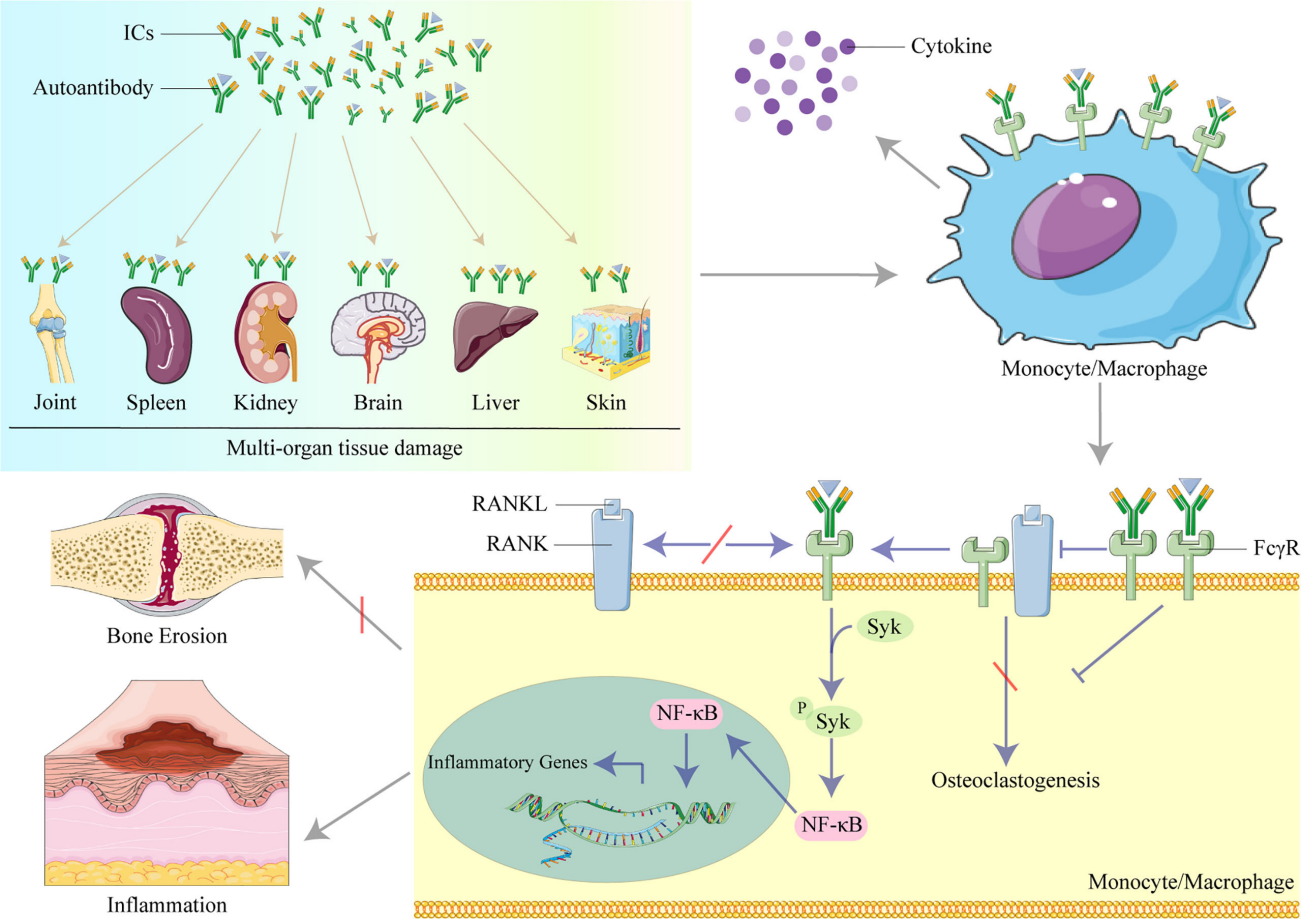
The role of IgG in organ and tissue damage in systemic lupus erythematosus (SLE). Lupus IgG and immune complexes (ICs) deposited in several organs and tissues cause multi-organ and tissue damage. IgG and ICs binding to Fc gamma receptors (FcγRs) activate monocytes/macrophages to secret cytokines. IgG inhibits osteoclastogenesis induced by the receptor activator of nuclear factor kappa B ligand (RANKL) to protect against bone erosion by binding to FcγRI and induces inflammation by activating the spleen tyrosine kinase (Syk)/nuclear factor kappa B (NF-κB) signal pathway to upregulate the transcription of inflammatory genes in monocytes/macrophages.

In this review, we summarize recent studies of the role of organ-deposited IgG in the pathogenesis of inflammation and organ and tissue damage in SLE, and potential therapeutic targets in the IgG/FcγRs signaling pathways, including studies investigating animal models, immune cells, cytokines (**Figure 1**)

## IgG deposited in tissues and organs

IgG and ICs have been found in the kidneys of SLE patients, and the most common autoantibodies identified are anti-dsDNA antibodies (18). IgM and IgG autoantibodies are deposited at the dermis–epidermis junction (29), forming so-called “lupus bands,” which can be detected using direct immunofluorescence (30, 31). Some autoantibodies, including anti-galectin 3 antibody, anti-acidic ribosomal protein P0 antibody (anti-RPLP0), and anti-Ro52 antibody, have been reported to be deposited in the skin and to be involved in the development of skin damage (32–34). We have demonstrated that IgG is deposited in the skin, liver, spleen, and joints of MRL/*lpr* mice (17, 23, 27, 28), and shown that the level of IgG deposited is correlated with the severity of tissue damage (17, 23, 27, 28). These findings suggest that tissue-deposited IgG may have an essential role in the pathogenesis of multi-organ and tissue damage in SLE.

## Serum-induced inflammation in organs and tissue

We investigated whether or not serum containing a high level of IgG induces tissue inflammation in order to determine the role of tissue-deposited IgG in the multi-organ and tissue damage that occurs in SLE. Through intra-organ injection of serum from lupus mice and SLE patients (lupus serum), we have established an animal model of SLE organ and tissue damage (17, 20–22, 24, 27, 28). We found that intra-organ injection of serum from SLE patients and lupus mice, but not from healthy humans and mice, induced tissue inflammation in the skin, liver, spleen, and joints, and that the severity of the inflammation was dose-dependent (17, 23, 27, 28). Using toll-like receptor 4 (TLR4)-deficient mice that were lipopolysaccharide (LPS) resistant, we established that organ and tissue inflammation could be induced by serum from SLE patients and lupus-prone mice, but not by LPS contamination (17, 27). We found that tissue inflammation caused by SLE serum appeared in the skin, liver, spleen, and joints a few hours after intra-organ injection, reached its peak after 3 days, and lasted for up to 14 days (17, 23, 27, 28).

Inflammatory skin reactions were induced by injecting serum from SLE patients into six different mouse strains: C57BL/6, SWISS, BALB/c, C3H/HeN, C3H/HeJ, and B-17 mice (23). Skin inflammation was induced by injection of serum only if the patients from which the serum was derived also exhibited skin inflammation and the severity of skin inflammation was not correlated with the severity of systemic disease (23); lupus serum had a synergistic effect with CpG DNA in inducing skin inflammation (23, 35).

Other studies have also shown that intracerebroventricular injection of lupus serum induces an inflammatory response (36–38). These studies suggest that lupus serum can cause organ and tissue inflammation.

## IgG plays a key role in inflammation induced by lupus serum

SLE is characterized by a high level of autoantibodies, particularly IgG. Many studies have shown that IgG is involved in the pathogenesis of organ and tissue inflammation and organ damage (17, 20–22, 24, 27, 28, 39, 40). We removed IgG from lupus serum to evaluate the role of IgG in the development of tissue inflammation. We found that the inflammation was significantly less severe in mice that received intra-organ injection of IgG-depleted lupus serum than in those injected with IgG-containing lupus serum (17, 23, 27, 28). The skin, liver, spleen, and joints could be directly inflamed by IgG isolated from lupus serum (17, 23, 27, 28). Furthermore, IgG extracted from lupus serum caused more severe inflammation than the same dose of IgG extracted from healthy serum (17, 23, 27, 28). The severity of inflammation was also related to the dose of lupus IgG (17, 23, 27, 28).

Recently, antibody glycosylation in autoimmune diseases has attracted attention, and the importance of Fc regions for inflammation development has been demonstrated (41, 42). In addition to the antigen-binding site, the level of antibody glycosylation may differ in serum from healthy individuals and SLE patients. *In vitro* experiments have revealed that microglia are activated by phagocytosis of IgG (36). In a study in which mice received intrasplenic injection of lupus serum or of IgG-depleted lupus serum, the numbers of germinal centers(GCs) and IgG-secreting plasmacytes incresased after 8 days in the whole lupus serum group. (28). IgG has also been shown to induce monocyte differentiation into dendritic cells (DCs) (24). In addition, intrahepatic injection of IgG has also been shown to increase levels of ALT alanine transaminase (ALT) and aspartate aminotransferase (AST) in serum and to cause accumulation of apoptotic hepatocytes around the region of inflammation (17).

In another study, the severity of IgG-induced skin inflammation was found not to be related to the titer of the anti-dsDNA antibody and anti-Ro antibody (23). In addition, it has been reported that injection of anti-DNA antibodies causes proteinuria and promotes lupus nephritis in mice only when the antibodies were bound to the basement membrane (43). These findings demonstrate that IgG plays a key role in tissue inflammation induced by lupus serum.

## FcγRI is important for the development of inflammation induced by SLE IgG

The effect of IgG is mediated by FcγR (44). FcγRI and FcγRIII are activating receptors, and FcγRII is the inhibitory receptor. The only inhibitory receptor, FcγRIIB, transmits its inhibitory signal through the immunoreceptor tyrosine-based inhibitory motif (ITIM) (45). Polymorphism of the genes encoding FcγRs has been reported and confirmed to be a heritable susceptibility factor for SLE (2, 3, 46). It has been reported that the *FCGR2B-I232T* genotype suppresses ligand binding, leading to low affinity for IgG (47). Because FcγRIIB is an inhibitory receptor, the low affinity for IgG contributes to a reduction in inhibitory signals, thus reducing inflammation, and alters the balance between inhibitory and activatory signals, which is associated with susceptibility to SLE (45, 47).

Similarly, polymorphism of the genes encoding activating receptors also influences susceptibility to SLE (2, 3, 47). FcγRI is the only high-affinity FcγR for IgG, and is usually expressed on the surface of monocytes/macrophages (48). The immunoreceptor tyrosine-based activation motif (ITAM) embedded in the intracellular structure of FcγR is responsible for signal transduction of IgG and ICs (44, 48). The expression of FcγRI, but not of FcγRII and FcγRIII, has been found to be increased in monocytes from SLE patients, and the level of FcγRI expression was found to be related to the SLE disease activity index (SLEDAI) (49).

We used FcγRI-deficient mice with IgG-induced inflammation to investigate the role of FcγRI in the inflammation induced by SLE (49). The inflammation induced by IgG was reduced in FcγRI-deficient mice compared with wild-type mice (49). In addition, the expression level of FcγRI was associated with IgG deposition and skin inflammation in MRL/*lpr* mice (37). Activation of the inflammatory signal pathway in monocytes/macrophages required FcγR expression (49). However, *in vitro* experiments have shown that IgG reduces the surface expression of FcγRI, but not of FcγRII and FcγRIII, on monocytes/macrophages (27). *N*-glycosylation of antibodies and IgG subclasses affects the binding affinity of antibodies to different Fc receptors (50). Abnormal glycosylation of autoreactive IgG may have a higher affinity to FcγRI, suggesting that the molecular modification of autoreactive IgG may influence its pathogenicity. Further work is required to identify the Fc region provided by which state of the SLE IgG.

## Monocytes/macrophages are required for inflammation induced by SLE IgG

Using various strains of cell-deficient and cell-depleted mice, we investigated the role of different types of immune cells in inflammation induced by IgG. Immunohistochemistry demonstrated that regions of tissue inflammation in the skin, kidneys, liver, and spleen are characterized by infiltration of a large number of monocytes/macrophages (17, 23, 27, 28, 51). For example, F4/80^+^ macrophages were identified in the red pulp of the spleen (28), liver macrophages (Kupffer cells) were seen in areas of hepatitis induced by SLE IgG (17), and large numbers of CD11b^+^ monocytes/macrophage and CD11c^+^ DCs were detected in sites of skin inflammation; however, CD3^+^ T cells and CD20^+^ B cells were absent (23, 52). The injection of lupus serum and IgG significantly reduced the inflammation caused by the depletion of monocytes/macrophages (17, 23, 27, 28).

Furthermore, skin inflammation induced by lupus IgG did not develop in *Csf1*-deficient mice lacking mature monocytes (21). *Rag 1*-deficient mice (which lack mature T and B cells but whose monocytes/macrophages are intact) were used to identify the role of T and B cells in IgG-induced inflammation (17, 24, 27). The lack of mature T and B cells did not affect the development of organ tissue inflammation induced by IgG (17, 24, 27). Neutrophil depletion by anti-Ly6G antibody injection did not affect the development of IgG-induced arthritis and dermatitis (20, 27). Another study showed that IgG induced lupus nephritis by activating macrophages (51).

In yet another study, IgG activated microglia to produce proinflammatory cytokines (53). In addition, polymorphism of the *ITGAM* (integrin subunit alpha M) gene (a gene associated with the activation of monocytes/macrophages) is associated with SLE susceptibility (11), and inflammation and organ damage in lupus-prone mice were suppressed, and splenic macrophages decreased, after treatment with the agonist of *ITGAM* (54). These findings indicated that monocytes/macrophages but not T cells, B cells, or neutrophils, play a crucial role in IgG-induced inflammation.

## TNF-α/TNFR1 plays a major role in the development of inflammation induced by SLE IgG

Many cytokines are involved in inflammation induced by IgG. Tumor necrosis factor alpha (TNF-α) is an important proinflammatory cytokine that is mainly secreted by macrophages and associated with SLE activity (55, 56). TNF-α has been found to be highly expressed in the kidneys and skin of SLE patients (57), and in the joints, skin, and kidneys of MRL/*lpr* mice (21, 27, 58). *In vitro* stimulation of macrophages with IgG has been found to cause IC to produce TNF-α (17, 28, 51),, and the severity of inflammation induced by IgG in skin, liver, spleen, and joints has been shown to be significantly reduced in mice with a TNF-α deficiency (17, 22, 27, 28). TNF exerts its effect through its receptors, such as TNFR1 and TNFR2. The skin inflammation induced by IgG was found to be reduced in mice with a TNFR1 deficiency, but not in those mice with a TNFR2 deficiency (24). Furthermore, we found that a deficiency of interleukin (IL) 1 (IL1) relieved inflammation induced by IgG by reducing the production of TNF-α (22). These results suggest that TNF-α/TNFR1 is crucial for inflammation induced by IgG in lupus serum. In addition, BAFF, produced by activated macrophages, was found in the region of inflammation to prolong the survival of macrophages in lupus nephritis (59, 60). In addition, IL-10, interferon alpha (IFN-α), and interferon gamma (IFN-γ) have been shown to increase CD64 expression in monocytes/macrophages (49, 61). In one study, a lack of macrophage migration inhibitory factor (MIF) attenuated infiltration of macrophages and alleviated lupus nephritis (62).

## Inhibitor of/FcγRs signaling pathway suppressed organ and tissue damage in lupus mice

Based on the findings from animal models of tissue inflammation induced by lupus IgG, the IgG/FcγRs signaling pathway is an important therapeutic target. Thus, we investigated whether an IgG/FcγRs signaling molecule inhibitor prevents and alleviates multi-organ and tissue damage in SLE. Spleen tyrosine kinase (Syk), a non-receptor tyrosine kinase of the Src family, plays a fundamental role in IgG/FcγRs signaling pathway conduction in autoimmune diseases (27, 63–65). FcγRI was required to activate Syk induced by SLE serum in macrophages (49). Syk inhibitors were used to treat lupus MRL/*lpr* mice, and have been found to prevent and treat damaged skin, kidneys, and spleen in MRL/*lpr* mice (28, 63). In addition, Syk inhibitors reduced inflammation and damage in the spleen, liver, and skin induced by injection of IgG from lupus serum (17, 23, 28). Intravenous immunoglobin (IVIG) is an anti-inflammation therapy used to treat various acute and chronic autoimmune diseases (66). Its anti-inflammation effect is due to the blocking of Fc receptors, which cannot bind with pathological autoantibodies (66). Glycosylation of the Fc region of IgG affects its function of inflammation modulation. The sialic acid-rich IgG fraction of IVIG has an improved anti-inflammatory activity (66). These results suggest that IgG/FcγRs signaling molecules may be a potential therapeutic target in multi-organ tissue damage of SLE.

## IgG protects against bone erosion in SLE arthritis

Bone erosion is an important feature in inflammatory arthritis, such as rheumatoid arthritis; however, it does not occur in SLE arthritis. The receptor activator of nuclear factor kappa B ligand (RANKL) induces monocytes/macrophages to separate into osteoclasts that mitigate bone erosion (26). FcγRI is a costimulatory molecule of RANK, the receptor for RANKL that activates macrophage differentiation into osteoclasts (14). We investigated the role of IgG in macrophage differentiation to understand the mechanism by which IgG from lupus serum induces arthritis without bone erosion (27). We found that IgG inhibited osteoclastogenesis induced by RANKL in a dose-dependent manner, and deficiency of FcγRII and FcγRIII did not influence the inhibitory effect of IgG in the formation of osteoclasts; both IgG from lupus serum and RANKL reduced the level of FcγRI (27). The inhibitory effect on osteoclastogenesis was greater at high doses than in at doses of IgG, and increasing doses of RANKL gradually blocked the inhibitory effects of lupus IgG. The stronger inhibitory effects of lupus IgG on RANKL-mediated osteoclastogenesis were presented in cells pre-treated for 24 hours when compared with cells treated with both RANKL and lupus IgG at the same time; at 24 hours after RANKL stimulation, the inhibitory effect of lupus IgG on osteoclastogenesis was eliminated (27). Based on these data, it appears that the relationship between IgG and RANKL is competition for FcγRI. IgG inhibits RANKL-induced osteoclastogenesis through competition for FcγRI binding. The binding of IgG to FcγRI may lead to a functional deficiency of FcγRI on the cellular membrane, which is required for RANKL-induced osteoclastogenesis. The competitive occupation of FcγRI by IgG may be exploited to develop therapeutic approaches to prevent bone destruction in autoimmune/inflammatory arthritis.

## Conclusion

Based on these results, we conclude that IgG plays a crucial role in developing organ and tissue damage in SLE and in protecting against bone erosion in SLE arthritis, and that the IgG/FcγRs signaling pathway is an important therapeutic target in SLE.

## Author contributions

Conception and design, G-MD; manuscript writing, WQ and TY; critical revision of the manuscript, G-MD; and, final approval of manuscript, all authors.

## Funding

This study was supported by the National Natural Science Foundation of China (NSFC) (G-MD, 82171786).

## Acknowledgments

**Figure 1** was modified from Servier Medical Art (http://smart.servier.com/).

## Conflict of interest

The authors declare that the research was conducted in the absence of any commercial or financial relationships that could be construed as a potential conflict of interest.

## Publisher’s note

All claims expressed in this article are solely those of the authors and do not necessarily represent those of their affiliated organizations, or those of the publisher, the editors and the reviewers. Any product that may be evaluated in this article, or claim that may be made by its manufacturer, is not guaranteed or endorsed by the publisher.
